# Diagnostic efficiency of metagenomic next-generation sequencing for suspected infection in allogeneic hematopoietic stem cell transplantation recipients

**DOI:** 10.3389/fcimb.2023.1251509

**Published:** 2023-09-13

**Authors:** Jiayu Huang, Yeqian Zhao, Chuanhe Jiang, Dongsheng Han, Zengkai Pan, Zilu Zhang, Luxiang Wang, Wei Chen, Su Li, Yanmin Zhao, Xiaoxia Hu

**Affiliations:** ^1^ State Key Laboratory of Medical Genomics, Shanghai Institute of Hematology, National Research Center for Translational Medicine, Shanghai Rui Jin Hospital, Shanghai Jiao Tong University School of Medicine, Shanghai, China; ^2^ Collaborative Innovation Center of Hematology, Shanghai Jiao Tong University School of Medicine, Shanghai, China; ^3^ Bone Marrow Transplantation Center, The First Affiliated Hospital, School of Medicine, Zhejiang University, Hangzhou, China; ^4^ Centre of Clinical Laboratory, The First Affiliated Hospital, School of Medicine, Zhejiang University, Hangzhou, China; ^5^ Department of Pulmonary and Critical Care Medicine, Ruijin Hospital, Shanghai Jiao Tong University School of Medicine, Shanghai, China; ^6^ Institute of Respiratory Diseases, Shanghai Jiao Tong University School of Medicine, Shanghai, China; ^7^ GoBroad Medical Institute of Hematology (Shanghai Center), Liquan Hospital, Shanghai, China

**Keywords:** metagenomic next-generation sequencing, conventional microbiological tests, diagnostic efficiency, clinical infection, immune events

## Abstract

**Introduction:**

Immunosuppression predisposes allogeneic hematopoietic stem cell transplantation (allo-HSCT) recipients to infection. Prompt and accurate identification of pathogens is crucial to optimize treatment strategies. This multi-center retrospective study aimed to assess the ability of metagenomic next-generation sequencing (mNGS) to detect causative pathogens in febrile allo-HSCT recipients and examined its concordance with conventional microbiological tests (CMT).

**Methods:**

We performed mNGS and CMT on samples obtained from 153 patients with suspected infection during allo-HSCT. Patients were grouped based on their neutropenic status at the time of sampling.

**Results:**

The mNGS test was more sensitive than CMT (81.1% vs. 53.6%, *P*<0.001) for diagnosing clinically suspected infection, especially in the non-neutropenia cohort. mNGS could detect fungi and viruses better than bacteria, with a higher sensitivity than CMT. Immune events were diagnosed in 57.4% (35/61) of the febrile events with negative mNGS results, and 33.5% (48/143) with negative CMT results (*P*=0.002). The treatment success rate of the targeted anti-infection strategy was significantly higher when based on mNGS than on empirical antibiotics (85% vs. 56.5%, *P*=0.004).

**Conclusion:**

The mNGS test is superior to CMT for identifying clinically relevant pathogens, and provides valuable information for anti-infection strategies in allo-HSCT recipients. Additionally, attention should be paid to immune events in patients with negative mNGS results.

## 1 Introduction

Allogeneic hematopoietic stem cell transplantation (allo-HSCT) is a potentially curative therapy for various hematological disorders (Copelan et al., 2019). However, this life-saving procedure poses considerable challenges to patients. Post-transplant immunodeficiency, including severe neutropenia, increases the risk of life-threatening infection, one of the main causes of non-relapse mortality (Neofytos, 2019). The intensive conditioning regimens and immunosuppressive agents used in graft-versus-host disease (GVHD) prophylaxis and treatment further contribute to prolonged immune reconstitution (Zeiser and Blazar, 2017; Gagelmann and Kroger, 2021; Martinez-Cibrian et al., 2021). However, infection signs during transplantation are usually covert and difficult to differentiate from other non-infectious events, such as GVHD. On the other hand, infection, particularly virus infection, play an important role in the occurrence of immune events (Khosla et al., 2018; Ghobadi et al., 2019). Therefore, infection after allo-HSCT is a markedly intricate and multifactorial, distinguishable from infection following chemotherapy, and is the leading risk factor in different phases resulting from different responsible pathogens infection (Sahin et al., 2016). Hence, rapid and accurate identification of causative pathogens is urgently needed to facilitate timely therapy.

Smear microscopy, polymerase chain reaction (PCR) and culture are the conventional microbiological tests (CMT) most commonly used to identify pathogens. However, these methods are relatively insensitive, and pathogen culture is time-consuming. In last 5 years, metagenomic next-generation sequencing (mNGS) has emerged as a powerful alternative method to overcome suchchallenges (Lu et al., 2020), as it can efficiently detect pathogenic microorganisms which are difficult to detect via CMT and distinguish etiologic microorganisms from background commensals with high efficiency and short turnaround time(Huang et al., 2020). However, the diagnostic value of mNGS has not been well evaluated for infection in the context of allo-HSCT, in which more than 80% of febrile events with negative CMT results. In this study, we evaluated the ability of mNGS to detect pathogens in febrile allo-HSCT recipients and examined its concordance with CMT.

## 2 Materials and methods

### 2.1 Study design

We retrospectively analyzed the medical records of patients who received allo-HSCT at Ruijin Hospital, the First Affiliated Hospital of Zhejiang University, and Liquan Hospital, GoBroad Medical Institute of Hematology (Shanghai Center) from April 2021 to January 2023. Institutional databases were retrospectively reviewed to extract demographic, clinical and genetic data. All procedures complied with the tenets of the Helsinki Declaration. The requirement for written informed consent was waived, owing to the non-interventional and retrospective nature of the study.

### 2.2 Infection prophylaxis after allo-HSCT

For herpes simplex virus (HSV) prophylaxis, acyclovir was given at the dose of 400 mg twice a day for 12 months after transplantation (Henze et al., 2022). For pneumocystis jirovecii pneumonia (PJP) prophylaxis, sulfamethoxazole was administrated at a dose of 480mg thrice a week from 2 months to 12 months (Maschmeyer et al., 2016). Between April 2021 and August 2022 (before the introduction of letermovir in China), 111 allo-HSCT recipients were treated with ganciclovir with 5mg/kg twice a day for 7 days prior to transplantation as cytomegalovirus (CMV) prophylaxis (Ljungman et al., 2019). After August 2022, 42 allo-HSCT recipients were treated with letermovir at a dose adjusted to calcineurin inhibitors for at least 3 months. For primary fungal prophylaxis, all patients received posaconazole oral suspension at an initial dose of 200mg thrice a day at the start of the conditioning regimens (Maertens et al., 2018). The efficacy of posaconazole treatment was determined based on therapeutic drug monitoring. The optimal serum trough concentration is>0.5mg/ml for prophylaxis (Chen et al., 2018). In case the trough concentration did not reach the prophylactic concentration, we prefer to increment the dosing frequency to 200mg every 4-6 hours, or switching to tablets or intravenous formulation. The duration of fungal prevention is up to 6 months for allo-HSCT with haploidentical donors (HID) or matched/mismatched unrelated donors (MUD/MMUD) and 3 months for allo-HSCT with matched sibling donors (MSD).

### 2.3 Standards for mNGS and CMT detection

The criteria for determining positive mNGS detection varied depending on the type of microbes being detected (Xu et al., 2023). A microbe was considered a positive result for bacteria and fungi when the stringently mapped read number (SMRN) at the species level was ≥3 and relative abundance at the genus level was >30%. A microbe was considered a positive result for viruses when the SMRN was ≥3. A microbe was considered positive for parasites when the SMRN was ≥100.

All patients underwent CMT as indicated by the treating physicians. Different specimens were collected for testing based on the type of suspected infection (i.e., blood samples, puncture fluids, BALF, and CSF). Positive CMT results was verified when bacteria, fungi were detected via smear microscopy, culture, and (1,3)-β-D-glucan test (G test), or galactomannan test (GM test). Viruses including CMV and Epstein-Barr virus (EBV), were verified with plasma twice a week via the quantitative real-time PCR. HSV1 was verified by PCR with oropharyngeal swab samples.”

### 2.4 Definition for mNGS and CMT results assessment

The diagnosis was made by an independent clinical committee comprising two hematologists, one pulmonologist and one radiologist. We comprehensively evaluated the clinical symptoms, radiological manifestations, CMT, and responses to anti-infection therapy to classify the final diagnosis into four categories: clinically significant infection, infection without clinical significance, immune events, mixed events of clinically significant infection and immune events. Also, the identification of final causative pathogens was made based on comprehensive consideration. The mNGS results was classified as definite, probable, possible, unlikely, or false-negative causes of infection. (1) Definite: microbes detected were consistent with those detected by CMT; (2) Probable: microbes detected were probably causative pathogens; (3) Possible: microbes detected showed potential to cause infection, but not as a common cause based on the consideration of clinical medical records; (4) Unlikely: microbes detected by mNGS were non-causative pathogens; (5) False negative: mNGS result was negative, but the case was diagnosed with infection. On the condition of “Definite”, “Probable” and “Possible”, mNGS results were judged as true positive, while on the condition of “Unlikely” they tended to be false positive. The positive predictive value (PPV) and negative predictive value (NPV) of mNGS were calculated as the ratio of true positive or negative mNGS detection to all practical mNGS detection. Initial empirical antibiotic therapy was performed within 24-48 h of clinical signs (Wang et al., 2020). Treatment success was defined as the resolution or reduction of clinical symptoms. CMV detection is defined as the detection and quantification of CMV DNA by either mNGS or PCR in plasma (Ljungman et al., 2017). The definition of clinically significant CMV infection requires clinical symptoms and CMV DNA viremia, necessitating the initiation of anti-CMV therapy by a treating physician (Chemaly et al., 2020).

### 2.5 Statistical analysis

Data analysis and visual representation of the results were performed using IBM SPSS Statistics for Windows, version 25.0 (IBM Corp., Armonk, N.Y., USA) and R 4.2.0 software (R Foundation for Statistical Computing, Vienna, Austria). Continuous and categorical variables were presented as medians and rang variables, as well as counts and percentages, respectively. Comparative analysis was conducted by Pearson χ2 test and Fisher exact test for discrete variables where appropriate. *P-value <*0.05 was considered to be statistically significant.

## 3 Results

### 3.1 Patient demographics and sample collection

**Figure 1:**
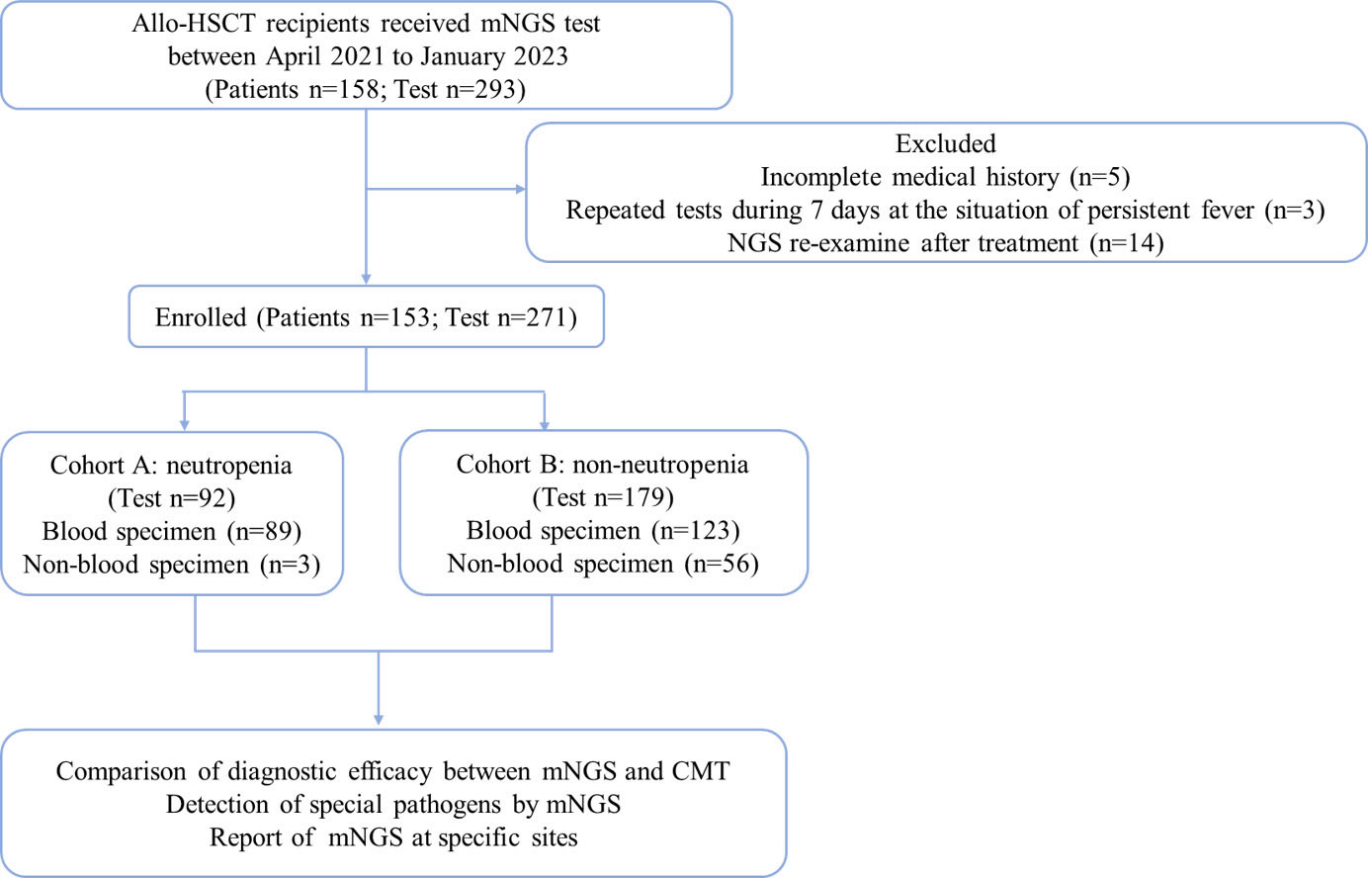
Flow chart of the study.

Based on the inclusion or exclusion criteria (**Figure 1**), we included 271 samples with parallel results for mNGS and CMT from 153 participants. General characteristics of 153 patients were summarized in **Table 1**. The median age was 47 (range, 15-69) years. We divided these samples into two cohorts based on neutropenic status at the time of sampling: cohort A (neutropenia [n=92]) and cohort B (non-neutropenia [n=179]) (**Table 2**). The most frequent mNGS sampling specimen was peripheral blood (78.2%), followed by BALF (10.7%), CSF (8.5%), and others (2.6%). Peripheral blood specimen was more common in cohort A than cohort B due to the non-invasive and easily accessible nature.

**Table 1:** Baseline characteristics description of 153 patients receiving allo-HSCT.

Characteristics		Total
		(n=153,%)
Age at allo-HSCT,years	Median(range)	47(15-69)
Gender	Male	85(55.6)
	Female	68(44.4)
Diagnosis	AML	88(57.5)
	MDS	20(13.1)
	ALL/LBL	32(20.9)
	MPAL	2(1.3)
	GS	2(1.3)
	Other diseases	9(5.9)
Conditioning regimens	MAC	133(86.9)
	RIC	20(13.1)
GVHD prophylaxis	ATG-based	54(35.3)
	PTCy-based	11(7.2)
	PTCy combined with ATG	75(49.0)
	CSA/MTX/MPA-based	13(8.5)
Donor Type	MUD/MMUD	14(9.2)
	MSD	15(9.8)
	HID	124(81.0)

allo-HSCT, allogeneic hematopoietic stem cell transplantation; AML, acute myeloid leukemia; MDS, myelodysplastic syndrome; ALL/LBL, acute lymphoblastic leukemia/lymphoma; MPAL, mixed-phenotype acute leukemia; GS, granulocytic sarcoma; MAC, myeloablative conditioning; RIC, reduced intensity conditioning; GVHD, graft-versus-host disease; ATG, antithymocyte globulin; PTCy, posttransplant cyclophosphamide; CSA,cyclosporin A; MTX, methotrexate; MPA, mycophenolic acid; MUD, matched unrelated donor; MMUD, mismatched unrelated donor; MSD, matched sibling donor; HID, haploidentical donor.

**Table 2:** mNGS samples.

Variable		Total
		(n=271,%)
Specimens	Peripheral blood	212(78.2)
	BALF	29(10.7)
	CSF	23(8.5)
	lung biopsy tissue	2(0.7)
	Urine	1(0.4)
	Mucosal secretions	2(0.7)
	Pleural effusion	2(0.7)
Sampling time	neutropenia	92(33.9)
	Non-neutropenia	179(66.1)
Time after allo-HSCT	0-12 days	79(29.2)
	12-30 days	67(24.7)
	30-90 days	67(24.7)
	>90 days	58(21.4)

BALF, bronchoalveolar lavage fluid; CSF, crebra-spinal fluid; allo-HSCT, allogeneic hematopoietic stem cell transplantation; mNGS, metagenomic next-generation sequencing.

### 3.2 Performance of mNGS and CMT for pathogen detection

We excluded 13 paired mNGS samples collected from different tissues in the same febrile event, and a comparison of the diagnostic results of mNGS with those of CMT methods was made for all 153 participants with 258 samples. The positive detection rate of mNGS was 76.4%, significantly higher than 43.8% of CMT (*P*<0.001). The positive detection rate was much higher with mNGS when compared with CMT in both cohort A (70.7% *vs.* 39.1% *P*<0.001) and cohort B (79.5% *vs.* 46.4% *P*<0.001). mNGS and CMT were concordant for 50 of 92 (54.3%) patients (kappa=0.128) in cohort A, and 91 of 166 (54.8%) patients (kappa=0.130) in cohort B (**Table 3**).

**Table 3:** Comparison of positive results and agreement among mNGS and CMT in different specimens.

			mNGS+ ^a^	mNGS-	Sensitivity (%)	Specificity (%)	PPV (%)	NPV (%)	kappa	agreement
blood specimen	cohort A(n=89)	CMT+	24	11	68.6	46.3	38.7	92.6	0.136	55.1
		CMT-	29	25						
	cohort B(n=123)	CMT+	47	6	88.7	32.9	48.5	88.5	0.197	56.9
		CMT-	47	23						
	Total(n=212)	CMT+	71	17	80.7	38.7	44.7	90.6	0.177	56.1
		CMT-	76	48						
	**cohort A *vs.* cohort B (*P*-value)**				0.019	0.128				
non-blood specimen	cohort A(n=3)	CMT+	1	0	100	0	33.3	0	0	33.3
		CMT-	2	0						
	cohort B(n=56)	CMT+	23	7	76.7	26.9	52.3	58.3	0.037	53.6
		CMT-	19	7						
	Total(n=59)	CMT+	24	7	77.4	25	51.1	58.3	0.025	52.5
		CMT-	21	7						
Total specimens	cohort A(n=92)	CMT+	25	11	69.4	44.6	38.5	92.6	0.128	54.3
		CMT-	31	25						
	cohort B(n=166)	CMT+	65	12	84.4	29.2	49.2	76.5	0.13	54.8
		CMT-	63	26						
	Total(n=258)	CMT+	90	23	79.6	35.2	45.7	83.6	0.139	54.7
		CMT-	94	51						
	**cohort A *vs.* cohort B (*P*-value)**				0.066	0.058				
	**blood *vs.* non-blood specimen (*P*-value)**				0.697	0.173				

a mNGS+: The NGS results were found to be consistent with those of CMT.

mNGS, metagenomic next-generation sequencing; CMT, conventional microbiological testing; PPV, positive predictive value; NPV, negative predictive value; cohort A, neutropenia; cohort B, non-neutropenia.

The detection rate of mNGS in peripheral blood specimens was 75.0%, higher than 41.5% with CMT methods (*P*<0.001). In non-blood specimens, the detection rates with mNGS and CMT were 79.7% and 54.2%, respectively (*P*=0.003).

### 3.3 Microbes landscape detected with mNGS and CMT

Regarding the clinical causative pathogens, bacteria accounted for 57.4% of infection in cohort A, followed by viruses (29.4%) and fungi (13.2%). In cohort B, viruses accounted for 61.1%, followed by bacteria (24.8%) and fungi (14.1%).

**Figure 2:**
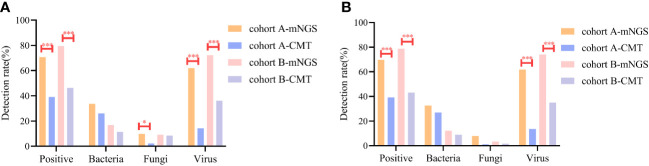
Comparison of detection sensitivities of the specific pathogens with mNGS and CMT in two cohorts. **(A)** Total specimens **(B)** Blood specimen. * *P*<0.05 *** *P*<0.001.

**Figure 3:**
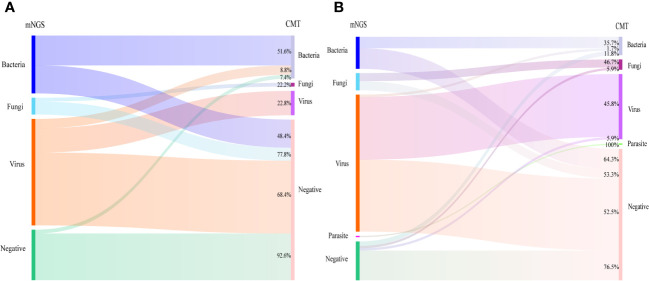
Summary of the relationship between mNGS and CMT results in cohort A **(A)** and cohort B **(B)**. Cohort A: neutropenia; Cohort B: non-neutropenia.

For bacteria, mNGS reached a 22.9% positivity rate compared with CMT (16.7%, *P*=0.077). There was no significant difference in the detection rates of bacteria using mNGS and CMT in cohorts A and B (**Figure 2A**). Similar results were observed when peripheral blood specimens were analyzed (**Figure 2B**). Among the 31 suspected bacterial infection cases in cohort A, mNGS results for 16 cases were consistent with CMT methods, with a concordance rate of 51.6% (16/31), and the concordance rate was 35.7% (10/28) in cohort B (**Figure 3**). Distribution of microbes detected by mNGS and CMT is shown in **Supplemental Figure S1**.

For suspected fungi, the detection rate with mNGS was similar to that of CMT (9.3% *vs.* 6.2% *P*=0.188) in all cases, but was higher than that of CMT in cohort A (9.8% *vs.* 2.2% *P*=0.03, **Figure 2A**). In cohort A and cohort B, 77.8% and 53.3% of the fungal infection verified by mNGS were not confirmed by CMT, respectively (**Figure 3**). Of the 14 cases of Aspergillus infection, thirteen cases (92.9%) were detected via mNGS, and only one case (A7, **Supplemental Table S1**) was detected via CMT method. A1 patient had respiratory symptoms and radiologic manifestation one month after mNGS-positive detection with peripheral blood in the neutropenic stage (**Supplemental Figure S2**).

**Figure 4:**
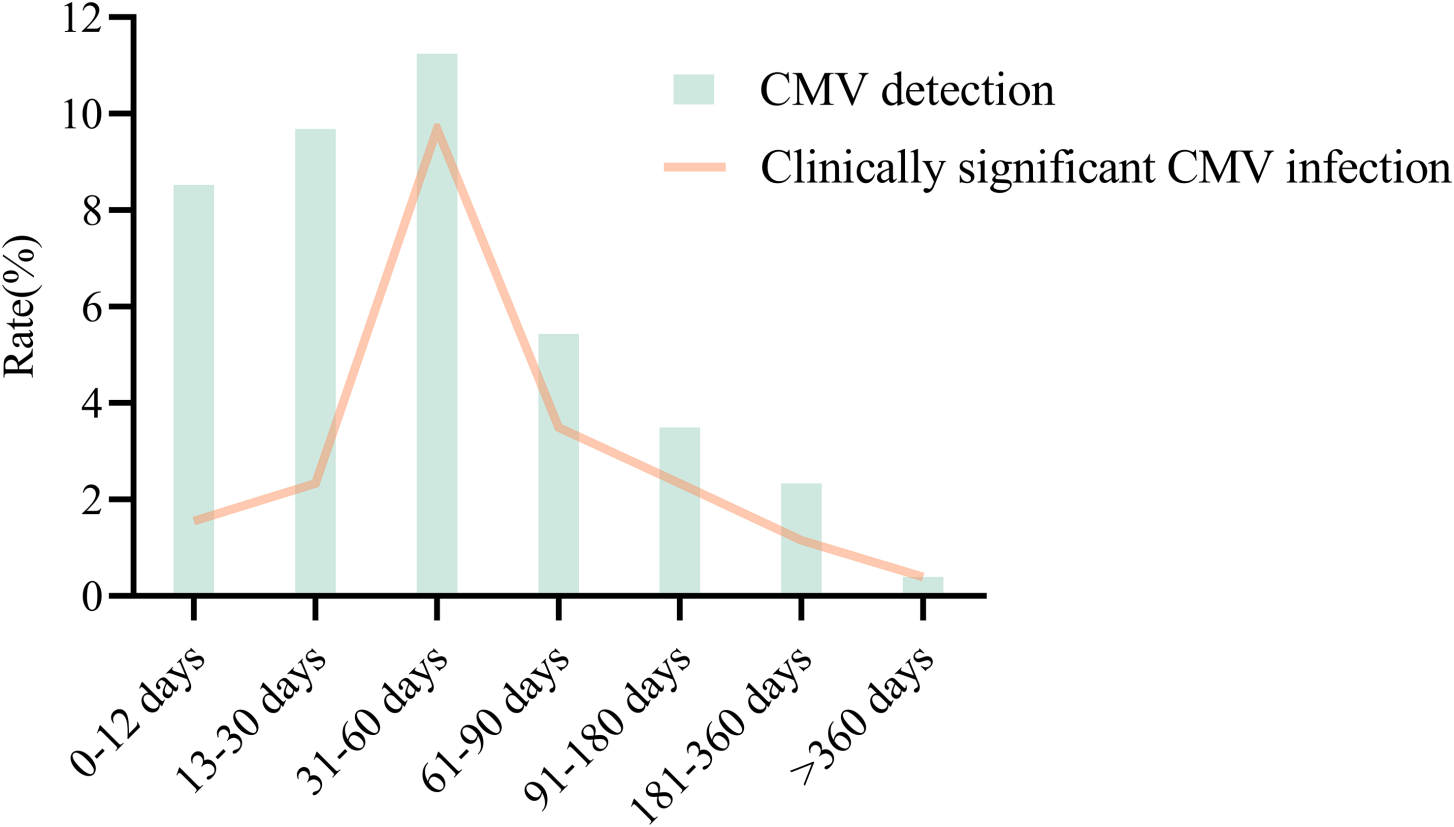
CMV detection and clinically significant CMV infection in different time periods after allo-HSCT.

More viruses had been identified via mNGS than via CMT because of its high sensitivity (cohort A: 62.0% *vs.* 14.1% *P*<0.001; cohort B: 72.3% *vs.* 36.1% *P*<0.001) (**Figure 2**). The concordance rates between mNGS and CMT for viral detection were 22.8% (13/57) in cohort A and 45.8% (55/120) in cohort B (**Figure 3**). CMV was the most frequently detected virus in both cohorts (cohort A: 33.7%; cohort B: 45.2%). As shown in **Figure 4**, the peak CMV detection rate using mNGS and the clinically significant CMV infection rate were concentrated between 30 and 60 days after allo-HSCT.

### 3.4 Microbes in non-blood specimens

In BALF specimens (29 cases, **Supplemental Table S2**), *Pneumocystis jirovecii* (*P. jirovecii*) and viruses constituted 79.3% of the microbes detected (**Supplemental Figure S3A**). Notably, *P. jirovecii* was detected only by mNGS. In patients with confirmed infectious pneumonia, the mNGS-positive detection rates were 89.3% (25/28 cases) and 64.2% for CMT (18/28 cases). In cases with PJP, the positive rate of the G test in the BALF was 83.3% (5/6 cases), and 12.5% in the peripheral blood (1/8). In 11 cases with positive BALF G/GM results, fungi were detected by mNGS in 6 cases (54.5%), including *Candida* in 16.7% (1 case) and *P. jirovecii* in 83.3% (5 cases). In 13 cases with Aspergillus infection verified by mNGS, five of which (38.5%, 5/13) was detected in BALF with mNGS test.

Viruses accounted for 69.6% of causative pathogens in suspected encephalitis (**Supplemental Table S3** and **Figure S3B**). Four patients (27.7%, C2, C5, C6, C18, C23) had simultaneous viremia when diagnosed with viral encephalitis, indicating that CSF testing is pivotal when encephalitis is suspected.

### 3.5 Diagnostic efficacies of mNGS and CMT on clinically suspected infection and immune events

A total of 168 patients (65.1%) were eventually diagnosed with clinically significant infection, 12 (4.7%) with infection but without clinical significance, 28 (10.9%) with mixed events, and 50 (19.4%) with immune events. The diagnostic capabilities of mNGS, CMT, and the combined protocols for different samples are shown in **Table 4**. The mNGS test yielded a higher sensitivity (77.4% *vs.* 52.9%, *P*<0.001) than the CMT for diagnosing clinically significant infection in peripheral blood samples, and the effect was more prominent in cohort B. In non-peripheral blood specimens, mNGS tended towards higher sensitivity than in peripheral blood specimens. (88.7% *vs.* 77.4%, *P*=0.075).

**Table 4:** The diagnostic efficacy of mNGS and CMT on clinical suspected infections.

			Sensitivity (%)	Specificity (%)	PPV (%)	NPV (%)
blood specimen	cohort A(n=89)	mNGS	74.2	70.4	74.2	70.4
		CMT	50.0	96.3	88.6	48.1
		**mNGS *vs.* CMT**	0.005	0.028		
		Combination protocol	83.9	70.4	81.3	76.0
		**Combination *vs.* mNGS**	0.186	1.000		
	cohort B(n=123)	mNGS	79.6	36.7	76.3	42.3
		CMT	54.8	96.7	96.2	41.4
		**mNGS *vs.* CMT**	<0.001	<0.001		
		Combination protocol	82.8	36.7	77.0	47.8
		**Combination *vs.* mNGS**	0.574	1.000		
	**mNGS cohort A *vs.* cohort B**		0.433	0.433	0.011	
	**combination cohort A *vs.* cohort B**		0.861	0.861	0.011	
	Total(n=212)	mNGS	77.4	52.6	75.5	56.6
		CMT	52.9	96.5	93.2	44.4
		**mNGS *vs.* CMT**	<0.001	<0.001		
		Combination protocol	83.2	52.6	78.7	62.5
		**Combination *vs.* mNGS**	0.199	1.000		
non-blood specimen	cohort A(n=3)	mNGS	100.0	/	100.0	/
		CMT	33.3	/	100.0	/
	cohort B(n=56)	mNGS	88.0	100.0	100.0	50.0
		CMT	54.0	100.0	90.0	23.1
		**mNGS *vs.* CMT**	<0.001	1.000		
		Combination protocol	96.0	100.0	98.0	85.7
		**Combination *vs.* mNGS**	0.1	1.000		
	Total(n=59)	mNGS	88.7	100.0	100.0	50.0
		CMT	52.8	100.0	90.3	21.4
		**mNGS *vs.* CMT**	<0.001	1.000		
		Combination protocol	96.2	100.0	98.1	85.7
		**Combination *vs.* mNGS**	0.133	1.000		
Total specimens	cohort A(n=92)	mNGS	75.4	70.4	75.4	70.4
		CMT	49.2	96.3	88.9	46.4
		**mNGS *vs.* CMT**	0.002	0.028		
		Combination protocol	84.6	70.4	82.1	76.0
		**Combination *vs.* mNGS**	0.188	1.000		
	cohort B(n=166)	mNGS	84.0	45.7	83.3	47.1
		CMT	55.7	94.3	94.8	37.1
		**mNGS *vs.* CMT**	<0.001	<0.001		
		Combination protocol	90.1	60.0	84.3	80.8
		**Combination vs. mNGS**	0.1	0.2		
	Total(n=258)	mNGS	81.1	56.5	80.7	57.4
		CMT	53.6	95.2	92.9	40.7
		mNGS *vs.* CMT	<0.001	<0.001		
		Combination protocol	88.3	54.8	83.6	66.7
		Combination *vs.* mNGS	0.050	0.857		
	**mNGS cohort A *vs.* cohort B**		0.148	0.052		
	**mNGS blood *vs.* non-blood specimen**		0.075	0.007		

mNGS, metagenomic next-generation sequencing; CMT, conventional microbiological testing; PPV, positive predictive value; NPV, negative predictive value; cohort A, neutropenia; cohort B, non-neutropenia.

Among the febrile events with negative mNGS results (61 samples), 35 patients (57.4%) were eventually diagnosed with immune events. Among the patients with negative CMT results (143 samples), 48 (33.5%) were diagnosed with immune events (*P*=0.002). The detailed immune events types are displayed in **Supplemental Table S4**.

### 3.6 mNGS in patients with negative CMT results

The spectrum of microorganisms in CMT negative cases were displayed in **Supplemental Figure S5**. In cohort A, 30 cases with negative CMT results were clinically diagnosed with an infection. Among the 30 cases, mNGS detected pathogens matched the final causative pathogens in 20 cases, including 10 cases of bacterial infection, 2 of fungal infection, 5 of viral infection and 3 of polymicrobial infection. In cohort B, mNGS verified 7 cases of bacterial infection, 3 of fungal infection, 26 of viral infection, and 5 of polymicrobial infection in infected participants with negative CMT results.

Lastly, we retrospectively reviewed the antibiotic regimen and clinical outcomes of the 86 patients. The overall treatment success rate (TSR) was 85% (34/40) for patients treated based on the mNGS results, and 56.5% for those receiving empirical therapy (26/46, *P*=0.004, **Supplemental Figure S4**).

## 4 Discussion

Developing targeted and precise antibiotic therapies without pathogenic evidence remains a substantial challenge in allo-HSCT recipients with underlying infection. In this retrospective study, we evaluated two approaches for microbial detection in allo-HSCT recipients: mNGS and CMT. Few studies have explored the clinical application of mNGS in allo-HSCT recipients (Liu et al., 2021; Zanella et al., 2021; Qu et al., 2022). Our study revealed the diagnostic efficacy superiority of mNGS in immunocompromised patients regardless of their neutropenic status.

The positivity rate of mNGS (76.4%) in our study was approximately 30% higher than that of CMT, consistent with a previous report (Xu et al., 2023). CMT is typically limited by its low coverage rate or restricted detection of a number of suspected microbes (Duan et al., 2021). Real-time metagenomics methods can identify pathogens faster than traditional culture-based techniques and potentially identify pathogens that cannot grow in cultures (Pendleton et al., 2017). Based on the final clinical diagnosis, our results showed that the sensitivity of mNGS (81.1%) was significantly higher than that of CMT (53.6%) for clinical infection requiring intervention. Other groups have reported similar results, highlighting the potential of mNGS to provide etiological evidence in CMT-negative patients (Miao et al., 2018; Wang et al., 2020). However, the specificity was lower than that reported previously (Miao et al., 2018), possibly because of false-positive results of low-quality virus readings. Nevertheless, the combination of mNGS and CMT increased diagnostic accuracy to 88.3%. We compared different antibiotic regimens with or without reference to mNGS results and demonstrated that mNGS had a positive impact on antibiotic adjustment decisions, especially in cases with negative CMT results. Additionally,immune events accounted for a higher proportion of mNGS-negative cases (52.6%) after allo-HSCT, indicating that negative mNGS might hint the presence of immune events, spanning acute GVHD, haplo-fever, engraftment syndrome (ES) and cryptogenic organizing pneumonia (COP, **Supplemental Table S4**). When physicians make a diagnosis of immune events and initiate steroid therapy, mNGS is recommended to rule out infection events, particularly for non-blood specimens.

Risk factors in the pre-engraftment phase include the presence of neutropenia, lasting for approximately 15–30 days (Sahin et al., 2016). Compared with patients with neutropenia after chemotherapy (Zhu et al., 2018), allo-HSCT recipients with neutropenia presented with a similar pathogen spectrum, with bacteria accounting for 57.4%. Viral infection was observed in all phases after allo-HSCT, but was the dominant causative pathogen in the non-neutropenia phase. In our study, mNGS could detect more virus than PCR. This may be explained by the technical limitations of PCR. The use of PCR in viral detection relies on genetic sequences of known pathogens and high pathogens loads. There are difficulties in detecting viruses with a copy number of less than 10^3^ copies. However, due to the high sensitivity of mNGS, it is easy to identify non-causative viruses that did not require clinical intervention. CMV infection is a serious complication after allo-HSCT. In our study, CMV reactivation was observed throughout the course of allo-HSCT. Previous studies have reported that CMV reactivation incidence ranges from 18% to 85%, with a median onset time of 32 to 41 days after allo-HSCT (Chang et al., 2020; Shen et al., 2022). It was documented that CMV reactivation after allo-HSCT was initiated approximately two weeks after engraftment (Talaya et al., 2020). Our results showed that clinically significant CMV infection was initiated before engraftment (< 12 days) in four cases. However, the incidence of clinically significant CMV infection peaked at 30 to 60 days.

Patients with neutropenia or those undergoing allo-HSCT are at high risk of fungi infection (Chien et al., 2019; Busca et al., 2021). In the present study, the fungi detection rate was 11.6%, which was lower than the 14.9% reported in another study (Qu et al., 2022). This difference may be due to the general prophylactic and pre-emptive therapy during allo-HSCT. It is worth noting that the mNGS test had higher sensibility than CMT on fungi-detection. Histopathological analysis is rarely performed in allo-HSCT recipients because of its invasive nature. In our study, nearly all patients (92.8%) were diagnosed with Aspergillosis using mNGS (including peripheral blood and BALF). Traditionally, Aspergillus cannot grow in peripheral blood, but its hyphae penetrate the air and blood barrier, thus eroding capillary endothelial cells and invading small arteries (Duan et al., 2021). Seven patients (7/14, 50%) were diagnosed with aspergillosis via mNGS with peripheral blood, six in cohort A (the neutropenic stage) and one in cohort B (non-neutropenic stage). *P. jirovecii* in BALF could only be detected with mNGS. The serum G test has been reported to be used as a reference index for PJP (Tasaka et al., 2007). In our study, of the six patients with PJP who received bronchial lavage for diagnosis, five patients (83.3%) were positive with the G test in the BALF sample, and only one patient (16.7% [1/6]) had a positive G test in peripheral blood.

This study has certain limitations. First, the sample size was small, and further studies with a larger cohort of allo-HSCT recipients are warranted. Second, the mNGS results could be influenced by many factors. In allo-HSCT procedures, fungi prophylaxis is generally performed for up to 3-6 months after transplantation. Prophylaxis could alter the atlas of causative pathogens detected by mNGS. Last, as we mentioned, in the non-neutropenia group, we were more predisposed to use non-blood samples for mNGS, which conferred a higher specificity than blood samples. Immune events were unique to allo-HSCT recipients. Whether mNGS would be used as a negative reference for immune events require further evaluation in future studies.

In summary, our study demonstrated that mNGS holds great potential for detecting causative pathogens of suspected infection and optimizing antibiotic treatment, through comparing the clinical efficacy of mNGS and CMT in allo-HSCT recipients. Further, we analyzed the differences on causative pathogen spectrum in different phases. Similar to the pathogen spectrum in patients with neutropenia after intensive chemotherapy, bacteria were most frequently detected in the neutropenic stage, while viruses were the dominant pathogens in the non-neutropenic stage. Immune events were unique for allo-HSCT recipients, with haplo-fever being commonly observed in the pre-engraftment phase, and GVHD in the post-engraftment phase. Therefore, immune events should be considered in patients with negative mNGS results.

## Data availability statement

The raw data supporting the conclusions of this article will be made available by the authors, without undue reservation.

## Ethics statement

Ethical approval and written informed consent for participation was not provided for this study on human participants because this study is a non-interventional and retrospective study and all procedures complied with the tenets of the Helsinki Declaration.

## Author contributions

WC, SL, YMZ, and XH designed the study, wrote and revised the manuscript. JH, YQZ, and CJ were involved in collecting, analyzing or interpreting research data and writing the manuscript. DH, ZP, ZZ, and LW analyzed research data. All authors contributed to the article and approved the submitted version.
