# Supplementary material for: Diagnostic efficiency of metagenomic next-generation sequencing for suspected infection in allogeneic hematopoietic stem cell transplantation recipients
Source: Front Cell Infect Microbiol. 2023 Sep 13;13:1251509. doi: 10.3389/fcimb.2023.1251509 (PMC10533937; doi:10.3389/fcimb.2023.1251509)

Supplemental Figure S1

Detection of specific microorganisms. (A) The number of subjects in whom each microbe was detected. (B) Distribution of microbes detected by mNGS and CMT in two cohorts. The top five causative bacterial pathogens were Klebsiella pneumoniae, Staphylococcus aureus, Enterococcus faecium, Streptococcus, Pseudomonas aeruginosa. Pneumocystis jirovecii, Aspergillus flavus, Aspergillus nidulans, Aspergillus tamarii, Cunninghamella were only detected with mNGS method.

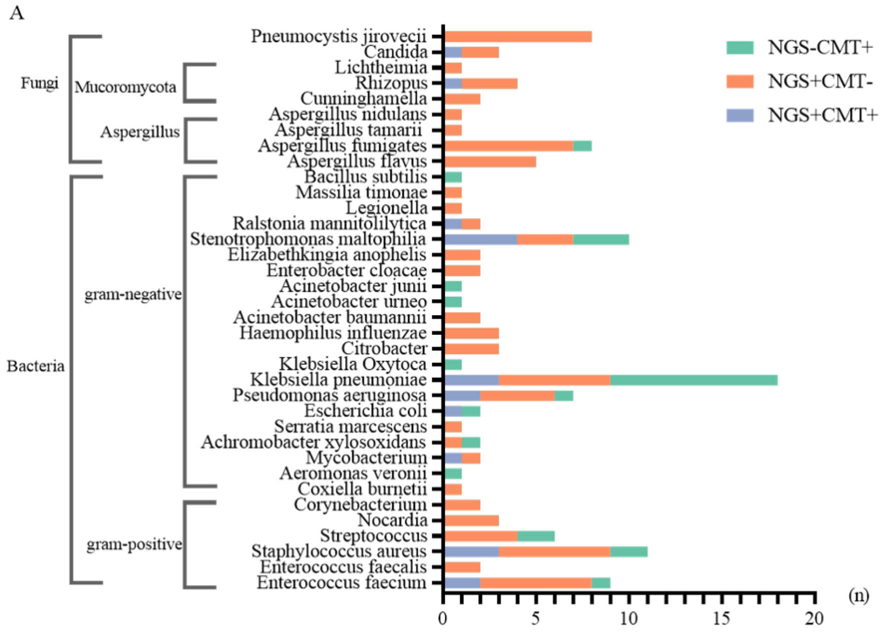

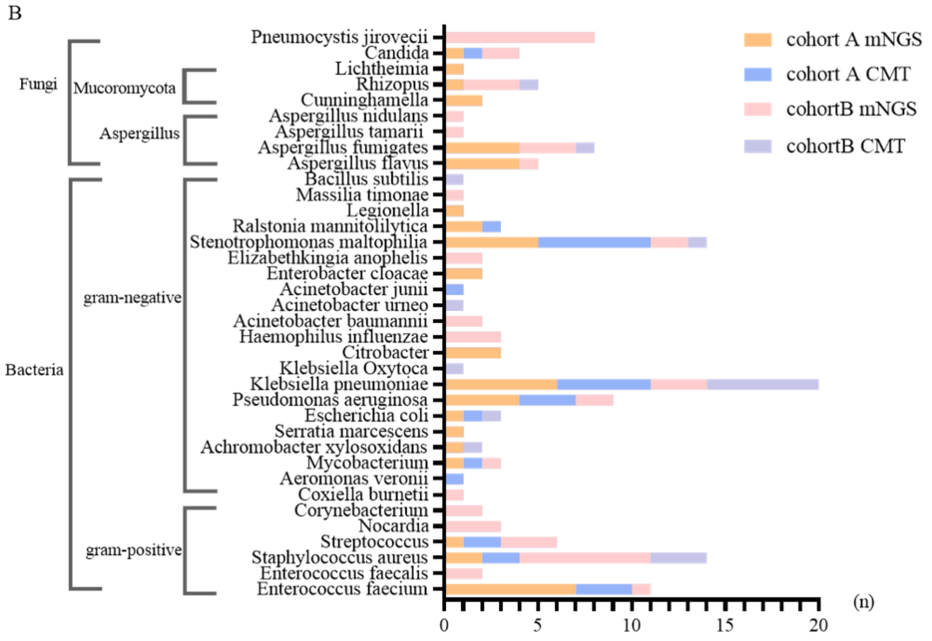

Supplemental Figure S2

Lung computed tomography (CT) manifestations of A1 patient with invasive aspergillus pulmonary infection. (A) CT sign when Aspergillus detected with mNGS (B) CT lesions at one month after mNGS detection of Aspergillus (C) CT sign at two months after mNGS detection of Aspergillus (one months after antifungal therapy). (D) CT sign after two-month antifungal therapy.

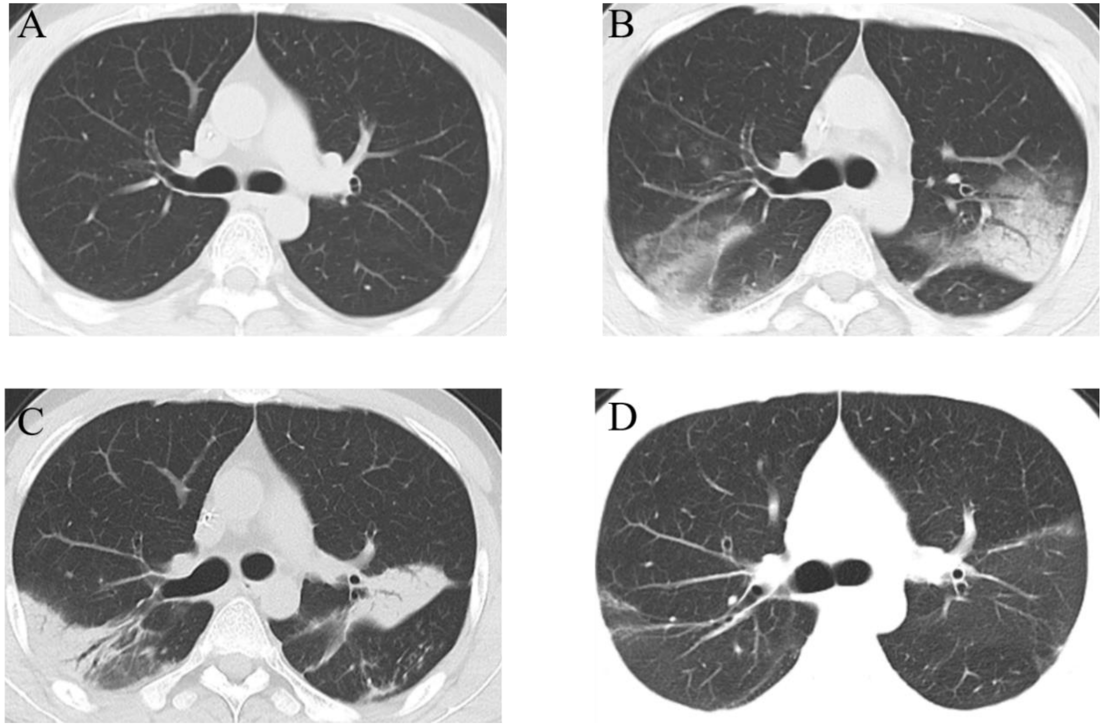

Supplemental Figure S3

Pathogens detected with mNGS in BALF and CSF. (A) BALF (n=29). (B) CSF (n=23).

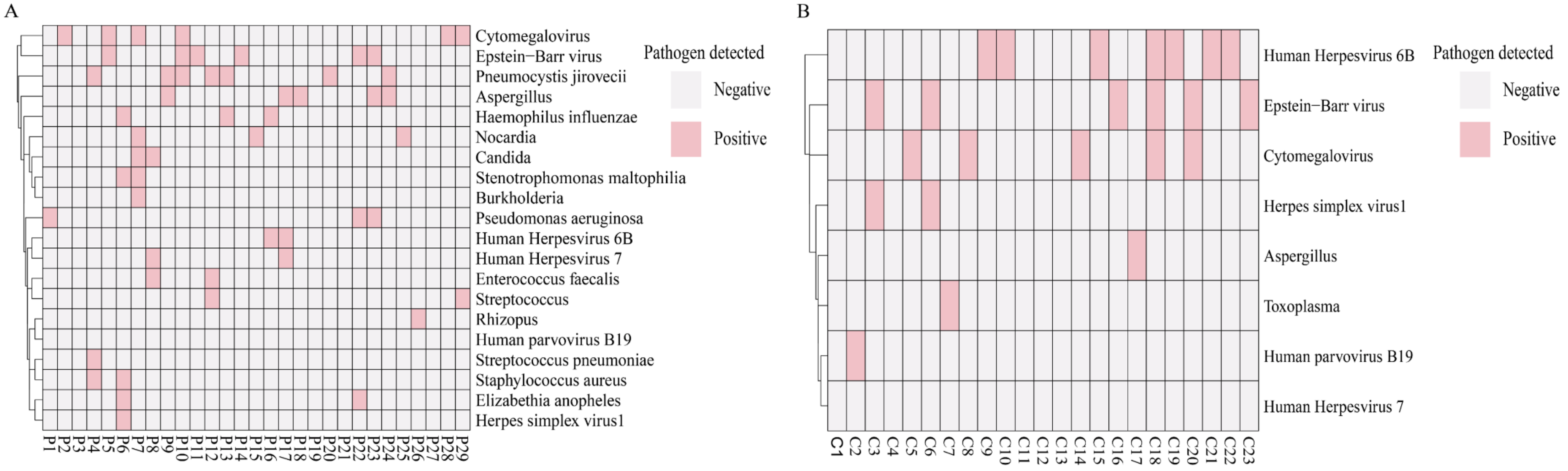

Supplemental Figure S4

Treatment results guided by mNGS or not mNGS results in patients with negative CMT results. TSR: treatment success rate.

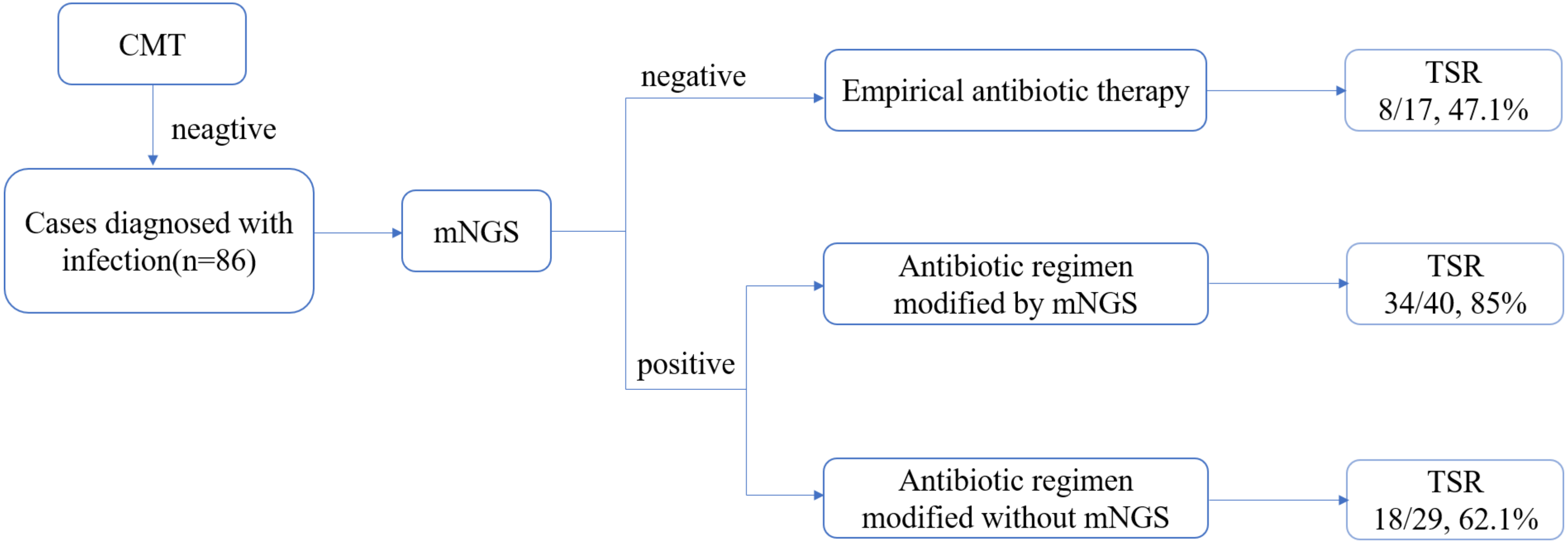

Supplemental Figure S5

Distribution of specific microorganisms in CMT-negative cases. HSV1: herpes simplex virus type 1; HHV6A: Human herpesvirus 6A; HHV6B: Human herpesvirus 6B; HHV7: Human Herpesvirus 7; B19: human parvovirus B19.

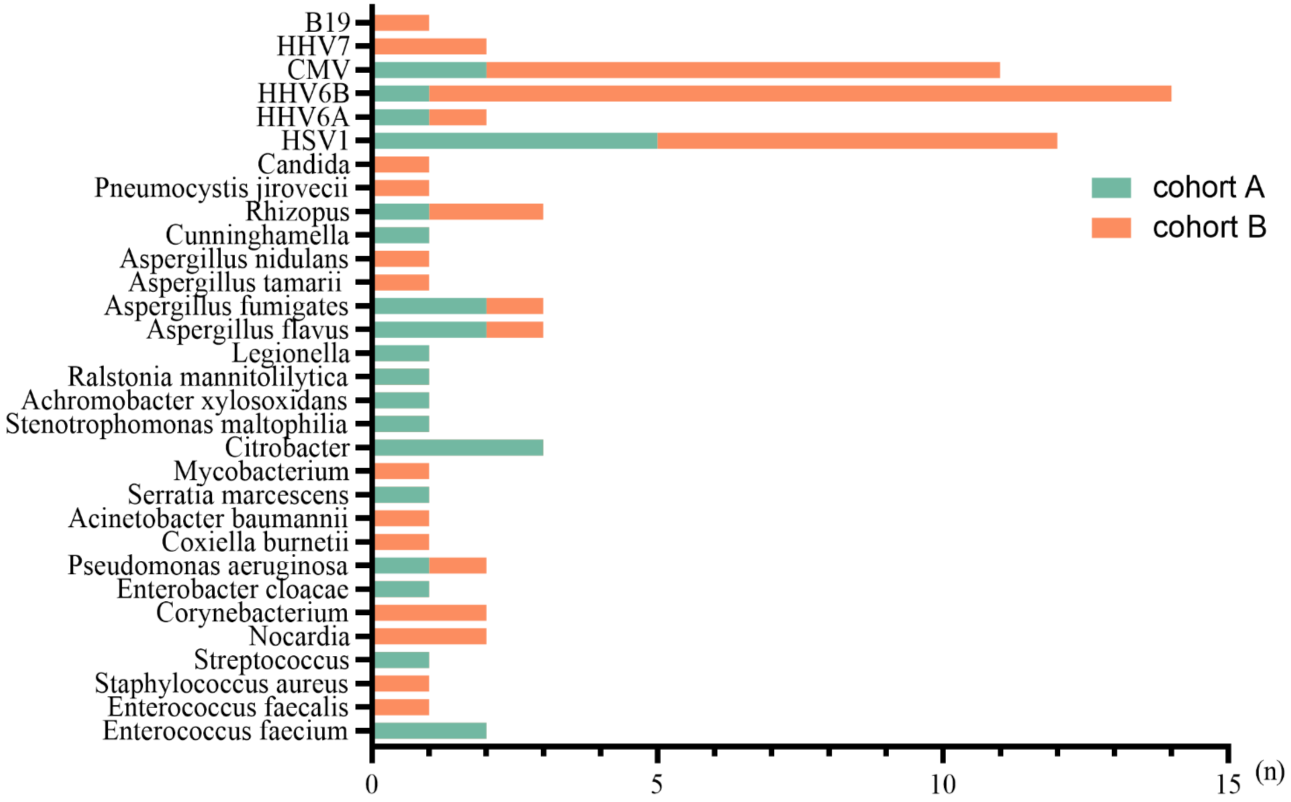

Supplement: Supplementary file 1 [file Image_1.pdf]
