# Supplementary material for: Diagnostic efficiency of metagenomic next-generation sequencing for suspected infection in allogeneic hematopoietic stem cell transplantation recipients
Source: Front Cell Infect Microbiol. 2023 Sep 13;13:1251509. doi: 10.3389/fcimb.2023.1251509 (PMC10533937; doi:10.3389/fcimb.2023.1251509)
Supplement: Supplementary file 2 [file Table_1.docx]

Supplemental Table S1. Description of the enrolled patients with aspergillus infection

| Patients | mNGS sampling cohort | mNGS sample | mNGS pathogen | Infection site | Blood G/GM | BALF G | non-BALF CMT |
| --- | --- | --- | --- | --- | --- | --- | --- |
|  |  |  |  |  |  |  |  |
| A1 | cohort A | Peripheral blood | Aspergillus flavus | unknown | negative |  | negative |
|  |  |  | HHV6B |  |  |  |  |
|  | cohort B | BALF | Aspergillus fumigatus | pulmonary | negative |  | negative |
|  |  |  | Aspergillus flavus |  |  |  |  |
|  |  |  | HHV7 |  |  |  |  |
|  |  |  | HHV6B |  |  |  |  |
| A2 | cohort A | Peripheral blood | Aspergillus flavus | unknown | negative |  | negative |
| A3 | cohort A | mucosal secretion | Aspergillus fumigatus | nasal mucosa | negative |  | negative |
|  |  |  | HSV1 |  |  |  |  |
| A4 | cohort B | BALF | Aspergillus fumigatus | pulmonary | negative | positive | negative |
|  |  |  | Pneumocystis jirovecii |  |  |  |  |
| A5 | cohort B | BALF | Aspergillus nestularis | pulmonary | negative |  | negative |
| A6 | cohort B | Peripheral blood | Aspergillus tamarii | pulmonary | negative |  | negative |
|  |  |  | HSV1 |  |  |  |  |
| A7 | cohort B | BALF | Pneumocystis jirovecii | pulmonary | negative | positive | Aspergillus fumigatus |
|  |  |  | Elizabethkingia anophelis |  |  |  | Elizabethkingia meningoseptica |
|  |  |  | EBV |  |  |  | EBV |
| A8 | cohort A | Peripheral blood | Klebsiella pneumoniae | unknown | negative |  | EBV |
|  |  |  | Mucoromycota |  |  |  |  |
|  |  |  | Aspergillus flavus |  |  |  |  |
|  |  |  | EBV |  |  |  |  |
| A9 | cohort A | Peripheral blood | Klebsiella pneumoniae | unknown | negative |  | EBV |
|  |  |  | Aspergillus flavus |  |  |  |  |
| A10 | cohort A | Peripheral blood | Mucoromycota | unknown | negative |  | negative |
|  |  |  | Aspergillus fumigatus |  |  |  |  |
| A11 | cohort A | BALF | Pseudomonas aeruginosa | pulmonary | negative |  | EBV |
|  |  |  | Aspergillus fumigatus |  |  |  | Exophiala dermatitidis |
|  |  |  | EBV |  |  |  |  |
| A12 | cohort B | BALF | Pneumocystis jirovecii | pulmonary | negative |  | negative |
|  |  |  | Aspergillus fumigatus |  |  |  |  |
| A13 | cohort A | Peripheral blood | Aspergillus fumigatus | unknown | negative |  | EBV |
| A14 | cohort B | CSF | Aspergillus flavus | encephalon, pulmonary | negative |  | EBV |
|  |  |  | Mucoromycota |  |  |  | Mucoromycota |

Abbreviation: mNGS: metagenomic next-generation sequencing; CMT: conventional microbiological testing; BALF:broncho alveolar lavage fluid; CSF:cerebro-spinal fluid;G: (1,3)-β-D-glucan test; GM: galactomannan test; HHV7: human herpesvirus 7; cohort A: neutropenia; cohort B: non-neutropenia; CMV:cytomegalovirus; EBV: epstein-barrvirus; HSV1: herpes simplex virus1; HHV6B: human herpes virus 6
