# Supplementary material for: Diagnostic efficiency of metagenomic next-generation sequencing for suspected infection in allogeneic hematopoietic stem cell transplantation recipients
Source: Front Cell Infect Microbiol. 2023 Sep 13;13:1251509. doi: 10.3389/fcimb.2023.1251509 (PMC10533937; doi:10.3389/fcimb.2023.1251509)
Supplement: Supplementary file 3 [file Table_2.docx]

Supplemental Table S2. The detailed information of patients receiving bronchoalveolar lavage

| Patients | BALF | | | |  | Peripheral blood | | | Sputum/Oral mucosal secretions |
| --- | --- | --- | --- | --- | --- | --- | --- | --- | --- |
|  | mNGS pathogen | CMT | | |  | mNGS pathogen | CMT | | CMT |
|  |  | Culture/smear | G/GM | virus PCR |  |  | G/GM | virus PCR | Culture |
| P1 | Pseudomonas aeruginosa | Negative |  | Negative |  | Negative | Negative | Negative | Negative |
| P2 | CMV | Negative | Negative | CMV |  |  | Negative | Negative | Negative |
| P3 | Negative | Negative | Positive | Negative |  |  | Negative | Negative | Negative |
| P4 | Streptococcus pneumoniae | Staphylococcus aureus | Negative | CMV |  |  | Negative | Negative | Negative |
|  | Staphylococcus aureus |  |  |  |  |  |  |  |  |
|  | Pneumocystis jirovecii |  |  | EBV |  |  |  |  |  |
| P5 | EBV | Negative | Positive | CMV |  |  | Negative |  | Klebsiella pneumoniae |
|  | CMV |  |  |  |  |  |  | Negative |  |
| P6 | Staphylococcus aureus | bacteria | Positive | HSV1 |  |  | Negative | Negative | Achromobacter xylosoxidans |
|  | Stenotrophomonas maltophilia |  |  |  |  |  |  |  |  |
|  | Haemophilus influenzae |  |  |  |  |  |  |  |  |
|  | Elizabethia anopheles |  |  | EBV |  |  |  |  | Paecilomyces variotii |
|  | HSV1 |  |  |  |  |  |  |  |  |
| P7 | Stenotrophomonas maltophilia | Fungi spore | Positive | Negative |  | CMV | Negative | CMV | Negative |
|  | Burkholderia |  |  |  |  |  |  |  |  |
|  | Nocardia |  |  |  |  |  |  |  |  |
|  | Candida albicans |  |  |  |  |  |  |  |  |
|  | CMV |  |  |  |  |  |  |  |  |
| P8 | Enterococcus faecalis | Negative |  | Negative |  |  | Negative | Negative | Negative |
|  | Candida |  |  |  |  |  |  |  |  |
|  | HHV7 |  |  |  |  |  |  |  |  |
| P9 | Aspergillus | Negative | Positive | Negative |  |  | Negative | Negative | Negative |
|  | Pneumocystis jirovecii |  |  |  |  |  |  |  |  |
| P10 | Pneumocystis jirovecii | Negative |  | Negative |  | CMV | Positive | Negative | HSV1 |
|  | CMV |  |  |  |  | EBV |  |  |  |
|  | EBV |  |  |  |  | Pseudomonas aeruginosa |  |  |  |
| P11 | EBV | Klebsiella pneumoniae, | Positive | EBV |  |  | Positive | Negative | Negative |
|  |  | Fungi spore |  |  |  |  |  |  |  |
|  |  | bacteria |  |  |  |  |  |  |  |
| P12 | Enterococcus faecalis | bacteria | Positive | Negative |  |  | Negative | Negative | Negative |
|  | Streptococcus | Pneumocystis jirovecii |  |  |  |  |  |  |  |
|  | Pneumocystis jirovecii | Fungi spore |  |  |  |  |  |  |  |
| P13 | Haemophilus influenzae | Klebsiella pneumoniae | Positive | Negative |  |  | Negative | Negative | Negative |
|  | Pneumocystis jirovecii |  |  |  |  |  |  |  |  |
| P14 | EBV | Negative |  | Negative |  |  | Positive | EBV | Negative |
| P15 | Nocardia | Negative | Negative | Negative |  |  | Negative | Negative | Negative |
| P16 | Haemophilus influenzae | Proteus mirabilis |  | Negative |  | HHV6B | Negative | Negative | Negative |
|  | HHV6B |  |  |  |  |  |  |  |  |
| P17 | Aspergillus | Negative |  | Negative |  |  | Negative | Negative | Negative |
|  | HHV7 |  |  |  |  |  |  |  |  |
|  | HHV6B |  |  |  |  |  |  |  |  |
| P18 | Aspergillus | Negative |  | Negative |  |  | Negative | Negative | Negative |
| P19 | Negative | Negative | Negative | Negative |  |  | Negative | EBV | Negative |
| P20 | Pneumocystis jirovecii | Negative | Positive | EBV |  |  | Negative | Negative | Negative |
| P21 | Negative | Negative | Positive | Negative |  |  | Negative | Negative | Negative |
| P22 | Elizabethia anopheles | Aspergillus fumigatus | Positive | EBV |  |  | Negative | Negative | Negative |
|  | Pneumocystis jirovecii |  |  |  |  |  |  |  |  |
|  | EBV | bacteria |  |  |  |  |  |  |  |
| P23 | Pseudomonas aeruginosa | Negative |  | Negative |  |  | Negative | EBV | aspergillus |
|  | Aspergillus |  |  |  |  |  |  |  |  |
|  | EBV |  |  |  |  |  |  |  |  |
| P24 | Pneumocystis jirovecii | Negative |  | Negative |  |  | Negative | Negative | Negative |
|  | Aspergillus |  |  |  |  |  |  |  |  |
| P25 | Nocardia | Negative |  | Negative |  |  | Negative | Negative | Negative |
| P26 | Rhizopus | Rhizopus |  | Negative |  |  | Negative | EBV | Negative |
| P27 | Negative | Negative |  | Negative |  |  | Negative | Negative | Negative |
| P28 | CMV | Negative |  | Negative |  |  | Negative | Negative | Negative |
| P29 | CMV | Negative | Negative | Negative |  |  | Negative | CMV | Negative |
|  | Streptococcus |  |  |  |  |  |  |  |  |

Abbreviation:BALF:broncho alveolar lavage fluid; mNGS: metagenomic next-generation sequencing; CMT: conventional microbiological testing; PCR: polymerase chain reaction; HHV7: human herpesvirus 7; HHV6B: human herpesvirus 6B; CMV:cytomegalovirus; EBV: epstein-barr virus; HSV1: herpes simplex virus1; G: (1,3)-β-D-glucan test; GM: galactomannan test; G+: Gram-positive bacteria; G-: Gram-negative bacteria
