# Supplementary material for: Diagnostic efficiency of metagenomic next-generation sequencing for suspected infection in allogeneic hematopoietic stem cell transplantation recipients
Source: Front Cell Infect Microbiol. 2023 Sep 13;13:1251509. doi: 10.3389/fcimb.2023.1251509 (PMC10533937; doi:10.3389/fcimb.2023.1251509)
Supplement: Supplementary file 4 [file Table_3.docx]

Supplemental Table S3. mNGS and CMT pathogens in suspected encephalitis

| Patients | CSF mNGS pathogen | Concomitant blood mNGS | CMT |  |
| --- | --- | --- | --- | --- |
|  |  |  |  |  |
| C1 | Negative |  | Negative |  |
| C2 | Parvovirus B19 | Parvovirus B19 | Negative |  |
| C3 | HSV1 |  | Negative |  |
|  | EBV |  |  |  |
| C4 | Negative | Negative | Negative |  |
| C5 | CMV |  | CMV |  |
| C6 | EBV |  | EBV |  |
|  | HSV1 |  |  |  |
| C7 | Toxoplasma gondii | Toxoplasma gondii | Toxoplasma gondii IgM+ |  |
| C8 | CMV |  | Negative |  |
| C9 | HHV6B |  | Negative |  |
| C10 | HHV6B |  | Negative |  |
| C11 | Negative |  | Negative |  |
| C12 | Negative |  | Negative |  |
| C13 | Negative |  | Negative |  |
| C14 | CMV |  | Negative |  |
| C15 | HHV6B |  | Negative |  |
| C16 | EBV |  | Negative |  |
| C17 | Aspergillus |  | EBV |  |
| C18 | EBV |  | EBV |  |
|  | HHV6B |  |  |  |
|  | CMV |  |  |  |
| C19 | HHV6B |  | Negative |  |
| C20 | EBV |  | Negative |  |
|  | CMV |  |  |  |
| C21 | HHV6B |  | Negative |  |
| C22 | HHV6B |  | Negative |  |
| C23 | EBV |  | EBV |  |

Abbreviation: CSF: cerebro-spinal fluid; mNGS: metagenomic next-generation sequencing; CMT: conventional microbiological testing; PCR: polymerase chain reaction; HHV7: human herpesvirus 7; HHV6B: human herpesvirus 6B; CMV: cytomegalovirus; EBV: epstein-barr virus; HSV1: herpes simplex virus1
