# Supplementary material for: Diagnostic efficiency of metagenomic next-generation sequencing for suspected infection in allogeneic hematopoietic stem cell transplantation recipients
Source: Front Cell Infect Microbiol. 2023 Sep 13;13:1251509. doi: 10.3389/fcimb.2023.1251509 (PMC10533937; doi:10.3389/fcimb.2023.1251509)
Supplement: Supplementary file 5 [file Table_4.docx]

Supplemental Table S4. mNGS and CMT pathogens in suspected encephalitis

| mNGS | mNGS sampling cohort | events | n(%) | immue events | n(%) |
| --- | --- | --- | --- | --- | --- |
|  |  |  |  |  |  |
| negative | cohort A (n=27) | immune events | 19(70.4) | ES | 3(15.8) |
|  |  |  |  | haplo-fever | 16(84.2) |
|  |  | mixed events | 4(14.8) | ES | 1(25) |
|  |  |  |  | haplo-fever | 3(75) |
|  | cohort B(n=34) | immune events | 16(47.1) | ES | 3(18.7) |
|  |  |  |  | COP | 1(6.3) |
|  |  |  |  | acute GVHD | 5(31.2) |
|  |  |  |  | haplo-fever | 1(6.3) |
|  |  |  |  | unexplained fever | 3(18.7) |
|  |  |  |  | immune encephalitis | 3(18.7) |
|  |  | mixed events | 2(5.8) | acute GVHD | 1(50) |
|  |  |  |  | haplo-fever | 1(50) |
| virus | cohort A (n=28) | immune events | 3(10.7) | ES | 2(66.7) |
|  |  |  |  | unexplained fever | 1(33.3) |
|  |  | mixed events | 3(10.7) | acute GVHD | 2(66.7) |
|  |  |  |  | haplo-fever | 1(33.3) |
|  | cohort B (n=95) | immune events | 12(12.6) | ES | 6(50) |
|  |  |  |  | acute GVHD | 6(50) |
|  |  | mixed events | 13(12.6) | ES | 3(23.1) |
|  |  |  |  | COP | 3(23.1) |
|  |  |  |  | acute GVHD | 2(15.4) |
|  |  |  |  | unexplained fever | 2(15.4) |
|  |  |  |  | immune encephalitis | 3(23.1) |
| Other | cohort A(n=37) | mixed events | 2(5.4) | ES | 1(50) |
|  |  |  |  | acute GVHD | 1(50) |
|  | cohort B(n=37) | mixed events | 4(10.8) | ES | 1(25) |
|  |  |  |  | COP | 2(50) |
|  |  |  |  | unexplained fever | 1(25) |

Abbreviation: mNGS: metagenomic next-generation sequencing; cohort A: neutropenia; cohort B: non-neutropenia; ES: engraftment syndrome; GVHD: graft-versus-host disease; COP: cryptogenic organizing pneumonia
